# Hospital costs of immunopreventable diseases in the economically active population in Brazil

**DOI:** 10.1186/s12913-021-07029-4

**Published:** 2021-11-11

**Authors:** Élide Sbardellotto M. da Costa, Adriano Hyeda, Eliane M. C. P. Maluf

**Affiliations:** Federal University of Paraná (UFPR), General Carneiro Street, 181, Curitiba, Paraná 80.060-900 Brazil

**Keywords:** Employment, Communicable diseases, Vaccines, Unified health system

## Abstract

**Background:**

Immunopreventable diseases are a public health reality in Brazil and worldwide, a reality that is not exclusive to children, but affects the adult population.

**Objectives:**

Discriminating the total costs of hospitalizations from immunopreventable diseases in the population aged 20 to 59 years.

**Methods:**

A population, observational, descriptive, retrospective study was conducted with secondary information from DATASUS to discriminate the hospitalizations associated with immunopreventable diseases in Brazil and their care costs, within the Scope of the SUS, between 2008 and 2018, in the economically active population (20 to 59 years).

**Results:**

It was analyzed 127,746 hospitalizations for immunopreventable diseases, (27.92% of all hospitalizations) were observed in the adult population, totaled R$115,682,097.54 (29.72% of the total costs). Of this population studied, 51.48% were registered as male; 66.74% were associated with influenza disease; 16.05% to chickenpox/herpes zoster infection and 7.55% to acute hepatitis B infections. The trend analysis of the time series of hospitalizations in this population showed a stationary trend.

**Conclusions:**

The 127,746 hospitalizations could be avoided with immunization, and 127,746 workers who could be working and not hospitalized. There were also R$115,682,097.54 that could be invested in other public health needs, which became necessary for the treatment of preventable diseases.

## Introduction

The World Health Organization (WHO) estimates that a quarter of deaths in children under 5 years are caused by immunopreventable diseases [1–7]. According to international literature [8–10], a considerable proportion of health care is attributed to communicable diseases, one in six cases attended by primary care and about 128,000 hospitalizations (84% in public hospitals) were related to these conditions in 2010. Vaccination is important in the care of these diseases, sinceit makes it possible both to avoid their incidence and to their complications and sequelae [8]. Only basic sanitation and drinking water have greater public health benefits than vaccination [5, 6]. Vaccines prevent between 2 and 3 million deaths per year worldwide [10].

According to The U.S. Department of Health and Human Services National Vaccine Plan (NVP) [11], 2010, despite the notorious knowledge about the safety and efficacy of vaccines, vaccination coverage over 18 years remains low in the U.S. They estimate that only one influenza-preventable disease has a total cost (between cost of health care and loss of productivity) of $87 billion dollars per year. And it is known that communicable diseases in the adult population impact both the individuals who get sick and their families (because they belong to the chain of transmission), as well as to society (with increased care costs, productivity losses and absenteeism).

The relevance of immunization of a population lies not only in eradication (in the case of smallpox), local elimination of diseases (such as measles and poliohiitis) and control of morbidity, mortality or complications of individual diseases (estimated reduction of six million deaths annually worldwide [5]. Vaccination also positively interferes: in the prevention of secondary infections and cancers associated with preventable diseases; protection of inadequately vaccinated populations (concept of collective immunization); reduction of care costs (such as hospitalizations, use of antibiotics and possible antimicrobial resistances, surgeries, costs with intensive care units and wards); increase in life expectancy and decrease in infant mortality; enables travel and social mobility globally; protects against some types of bioterrorism; stimulates the global economy (reduces absenteeism, increases individual production by decreasing the illness of workers) and promotes social equity in the world [5].

Thus, it is in the public interest to collect care data in this context of potentially preventable diseases nationally. Other countries have done similar research with very relevant data on how successful Immunization Programs positively interfere in reducing care and rehabilitation costs, gaining productivity, reducing absenteeism, and indirect social impact with these diseases.

## Methods

### Area of study

In this manuscript, data referring to Brazil were chosen for analysis. According to data from the Brazilian Institute of Geography and Statistics (IBGE, 2019/2020 - https://cidades.ibge.gov.br/brasil/panorama and https://cidades.ibge.gov.br/brasil/pesquisa/53/0?ano=2020), Brazil has an estimated population in 2020 of 211,755,692 people, with a population density of 22.43 inhabitants/km2, with a predominance of the population in the age groups of 10 to 29 years, a predominance of the female population, with life expectancy at birth of 7 years more for females (80 years on average). It has a predominantly urban population, with GDP per capita of R$31,833.50 (year 2017) and Human Development Index (HDI) of 0.761 (79th position in the world in 2019 - http://hdr.undp.org/en/content/2019-human-development-index-ranking).

### Study design

A population, observational, descriptive, retrospective study was conducted with multiple groups and time series, with aggregated secondary data, through information provided by the information system website of the Department of the Unified Health System (DATASUS - http://www2.datasus.gov.br/DATASUS/index.php?area=02) [12]. The research methodology on the DATASUS website was established according to the tools available in the consultation system: through the following links: “Health Information (TABNET)”, “Epidemiological and Morbidity”; “Hospital Morbidity of the SUS (SIH/SUS)”; “General with place of hospitalization - from 2008”; “Brazil by Region and Federation Units”; Line = “Age group 1”; Column = “not active”, content = “Hospitalizations; Hospital Admission Authorizations (AIH) approved; Total cost; Cost of hospital services; Cost of professional services; Average AIH cost; Average hospitalization cost; Days stay; Average permanence; Deaths; Mortality rate”; available period from January 2008 to December 2018; Chapter of ICD 10 = “I Infectious and parasitic diseases”; list of morbidities / ICD 10 = “Neonatal tetanus and other tetanus; Diffrhyphtheria; Whooping. Yellow Fever; Meningococcal infections; Measles; Rubella; Mumps; Human Rage; Chickenpox / Herpes Zoster; Acute hepatitis B” (diseases chosen because they have preventive vaccines available in the National Vaccination Calendar of the Brazilian Ministry of Health).

According to the technical note regarding general morbidity by place of hospitalization from 2008 [12] explains: “The data available (in tabnet) come from the Hospital Information System of SUS-SIH/SUS, managed by the Ministry of Health, through the Department of Health Care, together with the State Health Departments and the Municipal Health Departments, being processed by DATASUS - Department of Informatics of the SUS, of the Executive Secretariat of the Ministry of Health. The hospital units participating in the SUS (public or private-conveniadas) send the information of hospitalizations made through the AIH - Hospital Admission Authorization, to municipal managers (if in full management) or state (for the other). This information is consolidated in DATASUS, forming a valuable Database, containing data from most hospital admissions in Brazil.” The total costs were analyzed with all the hospital care.

The inclusions’ criteria were: the population secondary data known and publicly available on the DATASUS website (as mentioned above) from January 2008 to December 2018, referring to selected diseases (Neonatal tetanus and other tetanus; Diffrhyphtheria; Whooping. Yellow Fever; Meningococcal infections; Measles; Rubella; Mumps; Influenza (influenza); Chickenpox; Acute hepatitis B). The exclusions’ criteria were: data not available for consultation on the DATASUS website, on previously selected diseases. The DATASUS system itself, through the TABNET tool, makes available information available aggregated according to the selection made in the online system, and it is not necessary to delete missing data in this model. The outcomes analyzed were: the total total costs of hospitalizations related to the immunopreventable diseases mentioned above, and their evolution and temporal tendency. As secondary outcomes analyzed: the description of the costs of hospitalizations related to the aforementioned immunopreventable diseases, broken down by geographic region, year, disease, age group, gender, and descriptive economic measures.

The variables analyzed were the immunopreventable diseases mentioned above, year, age group, gender and economic variables. The socio-demographic data were tabulated and evaluated by descriptive statistics (mean, standard deviation, median and percentages), by excel® (Microsoft Corp., United States version 2007), Stata® (StataCorpLP, College Station, United States version 14.0), and Epi info 7®, by the research team itself. For the continuous (numerical) variables, linear regression analysis was used in the cases of verification of the correlations of the economic variables of each immunopreventable disease. The time trends (Yt) of the economic variables in relation to hospitalizations, age groups and genders were also analyzed, defined by the equation of linear regression given by Y_t_ = b_0_ + b_1t_ + and _t_. In this expression, parameter b_0_ corresponds to a constant, b_1_ corresponds to the slope of the line, and t is a random error, by the Prais-Winsten method. When the Beta parameter was positive, the time series was considered increasing; when negative, was considered descending; and stationary when there was no significant difference between its cost and zero. To measure the rate of variation of the line that adjusts the points of the time series, the basic logarithmic transformation 10 of the coefficients (Y) was performed, as it contributes to the reduction of the heterogeneity of the variance of the residuals of the linear regression analysis [13–15].

## Results

Data were analyzed for 457,479 hospitalizations broken down by age groups, as described in the Technical Note of DATASUS system ^11^ “Age group 1 comprises: Under 1 year, 1 to 4 years, 5 to 9 years, 10 to 14 years, 15 to 19 years, 20 to 29 years, 30 to 39 years, 40 to 49 years, 50 to 59 years, 60 to 69 years, 70 to 79 years, 80 years and older and ignored age”. Data were analyzed for 457,479 hospitalizations registered in the datasus public system, from 2008 to 2018.

A total of 127,746 hospitalizations (27.92% of all hospitalizations) were observed for immunopreventable diseases in the adult population (between 20 and 59 years, also corresponding to the economically active population), according to Table 1, according to ibge classification. This population group had 127,746 hospitalizations (with maximum amounts of 85,254 hospitalizations, minimum of 59 hospitalizations in the analyzed period, mean of 11,613.27, median of 1302, SD of 25,220.0, with 95% CI +/− 138.29). Referring to the total costs attributed to all hospitalizations for immunopreventable diseases analyzed in this age group, totaled R$115,682,097.54 (29.72% of the total costs, with maximum costs of R$65,417,395.74 in the period analyzed, minimum of R$11,923.47, average of R$10,516,554.32, median R$2,129,763.89, SD R$19,194,833.93, with 95% CI +/−R$3497.84), according to Table 2.

**Table 1:** Description of hospitalizations for immunopreventable diseases researched in Brazil, broken down by disease and age group (from 20 to 59 years), from 2008 to 2018

Immunopreventable Disease	Ages			
	20–29 years	30–39 years	40–49 years	50–59 years
Mumps	494	281	176	133
Whooping cough	66	57	58	46
Diphtheria	105	92	96	141
Yellow fever	282	361	421	338
Influenza	23,456	20,177	19,677	21,944
Hepatitis B	1151	2065	3045	3390
Meningococcal disease	2476	2014	1741	1338
Rubella / German measles	27	17	7	8
Measles	128	68	37	32
Neonatal and accidental tetanus	179	260	411	452
Chickenpox/Herpes Zoster	4488	4456	4999	6556
TOTAL	32,852	29,848	30,668	34,378

Source: SUS Information System (DATASUS - TABNET), data updated in February 2021

**Table 2:** Description of the values related to hospitalizations for immunopreventable diseases researched in Brazil, broken down by disease and age group (from 20 to 59 years), in the period from 2008 to 2018

Immunopreventable Disease	Ages			
	20–29 years	30–39 years	40–49 years	50–59 years
Mumps	R$ 100,485.28	R$ 58,824.95	R$ 51,841.86	R$ 34,766.63
Whooping cough	R$ 65,585.32	R$ 58,387.86	R$ 94,306.22	R$ 93,301.75
Diphtheria	R$ 190,802.84	R$ 189,147.44	R$ 216,846.83	R$ 384,056.01
Yellow fever	R$ 642,186.04	R$ 423,175.01	R$ 570,689.54	R$ 493,713.30
Influenza	R$ 16,515,703.89	R$ 14,921,731.37	R$ 15,713,988.13	R$ 18,265,972.35
Hepatitis B	R$ 719,203.29	R$ 1,729,574.63	R$ 2,495,070.43	R$ 2,869,201.02
Meningococcal disease	R$ 4,425,265.05	R$ 4,192,447.05	R$ 3,951,200.20	R$ 3,395,157.39
Rubella / German measles	R$ 6131.60	R$ 2832.55	R$ 1295.10	R$ 1664.22
Measles	R$ 33,344.13	R$ 25,596.52	R$ 13,027.31	R$ 14,008.51
Neonatal and accidental tetanus	R$ 614,935.46	R$ 1,318,430.67	R$ 2,052,156.93	R$ 2,800,066.40
Chickenpox/Herpes Zoster	R$ 2,880,801.00	R$ 3,221,943.80	R$ 3,957,090.72	R$ 5,876,140.94
TOTAL	R$ 26,194,443.90	R$ 26,142,091.85	R$ 29,117,513.27	R$ 34,228,048.52

Source: SUS Information System (DATASUS - TABNET), data updated in February 2021

Of this population studied, from 2008 to 2018, 51.48% were registered as male and 48.52% female. 66.74% of hospitalizations in this age group were associated with influenza disease; 16.05% to chickenpox/herpes zoster infection and 7.55% to acute hepatitis B infections. Among the total costs of hospitalizations, 56.55% were attributed to hospitalizations for influenza, 13.80% to hospitalizations for meningococcal disease and 13.78% to hospitalizations for chickenpox / herpes zoster.

**Fig. 1:**
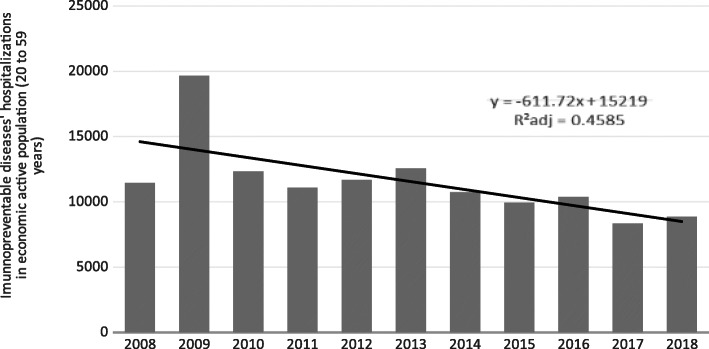
The graphic representation from the hospitalizations by imunnopreventable diseases analysed in the economic active population from Brazil, in the period from 2008 to 2018. Source: SUS Information System (DATASUS - TABNET), data updated in February 2021

**Fig. 2:**
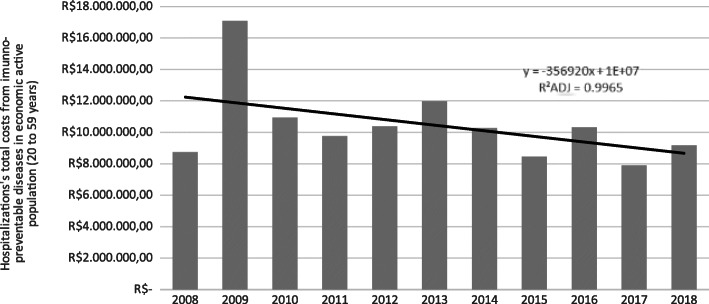
The graphic representation from the total costs from the hospitalizations by the imunnopreventable diseases analysed in the economic active population from Brazil, in the period from 2008 to 2018. Source: SUS Information System (DATASUS - TABNET), data updated in February 2021

The trend analysis of the time series of hospitalizations related to immunopreventable diseases, in the period from 2008 to 2018, emphasizing the reality of the economically active population (20 to 59 years) showed a stationary trend (without statistical significance) for hospitalizations (*p*-value 0.299 with 95% CI from − 0.043 to 0.125) and for the total costs related to these hospitalizations showed an decrease trend with statistical significance (*p*-value 0.004 with 95% CI from − 0.032 to − 0.008). This situation could be seeing in the Figs. 1 and 2, which show the trend lines for both analyzes: hospitalizations in the economic active population and the total costs from these hospitalizations in the 10 years (to 2008 from 2018). In those graphics it could be seeing the R^2^ analyzes that were R^2^ADJ = 0,4585 for the hospitalizations’ time series and the R^2^ADJ = 0,9965 for the total costs from theses hospitalizations. These R^2^ showed a strong relation between the dependent variable (Yt or hospitalizations and their costs) and the independent variable (βt or the years analyzed) in this analyze, with the statistical methods used.

## Method discussion

In the present study, data were observed regarding hospitalizations for immunopreventable diseases, that is, that present effective and widely available means of prevention: vaccines. International studies [16, 17] have already pointed to the importance of immunization in the adult population for various reasons, such as the impact on families, the community, as well as economic activities. In this economic reality, many implications can be signaled, such as the impact of the care costs of sick employees on health systems, whether public or private; the impact of the removal of sick employees (abscenteism) from preventable diseases on the production of companies. In the case of health workers, the need for adequate vaccination coverage is even more important, because it is not only individual immunity, but also not to transmit diseases to patients in the various health services, as studied in Italy in 2011 [17].

The data were organized from the database of the SUS Information Department website (DATASUS), using the Health Information System (TABNET), through thelink “Health Care, Hospital Morbidity of the SUS (SIH/SUS)”, by theoption “General, by place of hospitalization - from 2008”, in which data selections are made based on the classification by ICD 10 (per chapter of ICD 10 and by the “list of morbidities ICD 10” available on the site). Data were broken down by year of care, character of care, Chapter of ICD 10, age group, gender and region / Federation Unit. The economic data were broken down according to the data available on the site: total hospitalizations, approved AIHs, total cost of hospitalizations, cost of hospital services, cost of professional services, mean cost of AIHs, mean cost of hospitalization, days of stay, mean length of stay, total deaths recorded and mortality rate.

According to the technical note regarding general morbidity by place of hospitalization from 2008 [12] explains: “The data available (in tabnet) come from the Hospital Information System of SUS-SIH/SUS, managed by the Ministry of Health, through the Department of Health Care, together with the State Health Departments and the Municipal Health Departments, being processed by DATASUS - Department of Informatics of the SUS, of the Executive Secretariat of the Ministry of Health. The hospital units participating in the SUS (public or private-conveniadas) send the information of hospitalizations made through the AIH - Hospital Admission Authorization, to municipal managers (if in full management) or state (for the other). This information is consolidated in DATASUS, forming a valuable Database, containing data from most hospital admissions in Brazil.”

All studies based on public secondary databases have the limitation, already known, of underreporting and underreporting of the analyzed system itself, because these are dependent on the databases being fed by the employees responsible for the system. In the case of the SUS, these data are feeders in a decentralized manner and regionalized by States and Municipalities. However, despite the notorious underutilization of the system, these are the official data that are used for the development of public health policies in Brazil.

## Results discussion

In the case of this study, it was observed that the temporal trend analysis was stationary in the period from 2008 to 2018, both in the amounts of hospitalizations and in the total costs attributed to these hospitalizations. This may reflect the need for public health policies aimed at this population (such as educational campaigns in public transport and media; extended hours in health units; active search for peoplewho need vaccinesin schools, universities, companies, services and industries, among as many possible measures) to improve vaccination coverage and, consequently, reduce the incidence and hospitalizations of these diseases.

A reflection is needed: 127,746 hospitalizations for vaccine-preventable diseases are 127,746 hospitalizations that could be avoided, and 127,746 workers who could be working and not hospitalized. There were also R$115,682,097.54 that could be invested in other public health needs, which became necessary for the treatment of preventable diseases. Considering the volume of total costs flagged here, one can invest in raising awareness among health professionals about the importance of adequate vaccination coverage in this population, so when the adult population searches forhealth care, it is also oriented about its vaccination.

The main objective of this manuscript is not to determine the causal relationship for hospital costs for preventable diseases. The merit of this study is that it signals a reality that often goes unnoticed to the managers of the health system and the population: that diseases that are effectively preventable by vaccines still affect the Brazilian population, in a relevant amount, adding financial costs also relevant to the country’s public health system, regardless of gender and age (because here in this analysis we observe cases of immunopreventable diseases not only in children, but also in adults and the elderly, a reality observed internationally) [16–20]. These costs are not showing downward trends, but rather, they are proving stable over the time studied, even though vaccines are available free of charge to the entire population by the National Immunization Program for many years.

An opportunity for improvement that is observed is importance of employing awareness public and private campaigns for the importance of specific vaccination of this population group. It the companies and industries would invest in employee’s vaccination, they could avoid important cost with care costs, production loses and absenteeism. This awareness gains even more importance when observing the drop-in vaccination coverage globally during the 2020/2021 pandemic and falls in the notifications of other immunopreventable diseases [21], predisposing to the resurgence and increase in the incidence of immunopreventable diseases, a reality that is not exclusive to children, but affects the entire world population, regardless of age group or gender. This is a commitment that must be made by all countries, because immunizing the population is an investment to create a healthier, safer and more prosperous future for all, as the WHO [22, 23] guides.

## Conclusions

The 127,746 hospitalizations could be avoided with immunization, and 127,746 workers who could be working and not hospitalized. There were also R$115,682,097.54 that could be invested in other public health needs, which became necessary for the treatment of preventable diseases. An opportunity for improvement that is observed is importance of employing awareness public and private campaigns for the importance of specific vaccination of this population group. It the companies and industries would invest in employee’s vaccination, they could avoid important cost with care costs, production loses and absenteeism.

## Data Availability

The data that support the findings of this study are openly available in the information system website of the Department of the Unified Health System (DATASUS - http://www2.datasus.gov.br/DATASUS/index.php?area=02).

## Abbreviations

IBGE: Brazilian Institute of Geography and Statistics
CI: Confidence interval
DATASUS: Department of the Unified Health System
GDP: Gross Domestic Product
AIH: Hospital Admission Authorizations
HDI: Human Development Index
ICD 10: International Classification of Diseases and Health-Related Problems
SD: Standard deviation
SUS: Unified Health System
WHO: World Health Organization
